# NPRL-Z-1, as a New Topoisomerase II Poison, Induces Cell Apoptosis and ROS Generation in Human Renal Carcinoma Cells

**DOI:** 10.1371/journal.pone.0112220

**Published:** 2014-11-05

**Authors:** Szu-Ying Wu, Shiow-Lin Pan, Zhi-Yan Xiao, Jui-Ling Hsu, Mei-Chuan Chen, Kuo-Hsiung Lee, Che-Ming Teng

**Affiliations:** 1 Pharmacological Institute, College of Medicine, National Taiwan University, Taipei, Taiwan; 2 The Ph.D. program for Cancer Biology and Drug Discovery, College of Medical Science and Technology, Taipei Medical University, Taipei, Taiwan; 3 Beijing Key Laboratory of Active Substance Discovery and Drug ability Evaluation, Institute of Materia Medica, Chinese Academy of Medical Sciences and Peking Union Medical College, Beijing, China; 4 School of Pharmacy, College of Medicine, National Taiwan University, Taipei, Taiwan; 5 The Ph.D. Program for the Clinical Drug Discovery from Botanical Herbs, College of Pharmacy, Taipei Medical University, Taipei, Taiwan; 6 Graduate Institute of Pharmacognosy, Taipei Medical University, Taipei, Taiwan; 7 Natural Products Research Laboratories, Eshelman School of Pharmacy, University of North Carolina at Chapel Hill, Chapel Hill, North Carolina, United States of America; 8 Chinese Medicine Research and Development Center, China Medical University and Hospital, Taichung, Taiwan; Columbia University, United States of America

## Abstract

NPRL-Z-1 is a 4*β*-[(4″-benzamido)-amino]-4′-*O*-demethyl-epipodophyllotoxin derivative. Previous reports have shown that NPRL-Z-1 possesses anticancer activity. Here NPRL-Z-1 displayed cytotoxic effects against four human cancer cell lines (HCT 116, A549, ACHN, and A498) and exhibited potent activity in A498 human renal carcinoma cells, with an IC_50_ value of 2.38 µM via the MTT assay. We also found that NPRL-Z-1 induced cell cycle arrest in G1-phase and detected DNA double-strand breaks in A498 cells. NPRL-Z-1 induced ataxia telangiectasia-mutated (ATM) protein kinase phosphorylation at serine 1981, leading to the activation of DNA damage signaling pathways, including Chk2, histone H2AX, and p53/p21. By ICE assay, the data suggested that NPRL-Z-1 acted on and stabilized the topoisomerase II (TOP2)–DNA complex, leading to TOP2cc formation. NPRL-Z-1-induced DNA damage signaling and apoptotic death was also reversed by TOP2α or TOP2β knockdown. In addition, NPRL-Z-1 inhibited the Akt signaling pathway and induced reactive oxygen species (ROS) generation. These results demonstrated that NPRL-Z-1 appeared to be a novel TOP2 poison and ROS generator. Thus, NPRL-Z-1 may present a significant potential anticancer candidate against renal carcinoma.

## Introduction

Cancer is a leading cause of death worldwide. Many anticancer agents that target DNA are currently most effective clinically and lead to significant improvements in the survival of cancer patients when used along with drugs with different mechanisms [1]. DNA topoisomerases are recognized as important targets in anticancer drug discovery [2]–[5]; approximately 50% of current antitumor treatment regimens include at least one drug that acts as a topoisomerase inhibitor [6], [7].

Topoisomerases are essential nuclear enzymes that are involved in DNA supercoiling regulation and play key roles in transcription, replication, and chromosome segregation [8]–[11]. Two major classes of topoisomerases (types I and II) are distinguished by the number of DNA strands that they cleave and the mechanism by which they alter the topological properties of DNA [12], [13]. Topoisomerase I catalyzes supercoiled DNA unwinding by transiently creating a break in a single-stranded DNA (ssDNA) [14], whereas TOP2 creates DNA double-stranded breaks (DSBs) to allow the passage of a second double-stranded DNA through the transiently broken duplex [15]. There are two TOP2 isoforms, TOP2α (170 kDa) and TOP2β (180 kDa), in human cells. TOP2α levels peak at the G2/M phase and high levels of the same are expressed in proliferating tumor cells, whereas TOP2β levels do not significantly alter during cell cycle and is often present in both proliferating and differentiated cells [16]. Currently available TOP2-targeted drugs are classified as TOP2 poisons and catalytic inhibitors [17]. TOP2 poisons, such as etoposide (VP-16), doxorubicin, anthracyclines, ciprofloxacin, and amsacrine (*m*-AMSA), increase and stabilize covalent, TOP2-cleaved, DNA complexes, and thus block religation, enzymatic release, and, ultimately, induce apoptosis [18], [19]. Catalytic inhibitors inhibit the overall catalytic activity of TOP2, preferring not to stabilize the covalent TOP2–DNA cleavage complexes [20], [21]. In contrast, most TOP2 poisons frequently induce multidrug resistance and have dose-limiting toxicities, resulting in treatment failure after initial effective therapy [22], [23]. Moreover, they may trigger chromosomal translocations that can lead to a specific leukemia type by inducing DNA strand breaks [21], [24]. Therefore, considerable cancer research is the current focus to develop novel TOP2-targeted drugs in order to improve the current situation.

NPRL-Z-1, 4′-*O*-Demethyl-4*β*-[4″(benzyl _L_-alanyl-N-carbonyl)-anilino]-4 -desoxy-podophyllotoxin, is synthesized by Dr. Lee *et al.* (Natural Products Laboratory, University of North Carolina, Chapel Hill, NC, USA), and designed to enhance TOP2 inhibition, overcome drug resistance, and modulate water solubility of etoposide analogues by extending the bulky substituent at C7 [25] (It was compound 14 in the cited article). Here NPRL-Z-1 mechanisms associated with cell apoptosis in human renal cell carcinoma (RCC) A498 cells were first investigated, which showed that NPRL-Z-1 had better cytotoxicity against A498 cancer cells than etoposide and induced DNA damage through TOP2 inhibition. The data suggested that NPRL-Z-1 is a novel TOP2 poison and provides an alternative mechanism that can be exploited in RCC therapy.

## Materials and Methods

### Reagents and chemicals

NPRL-Z-1 was synthesized by Dr. Lee *et al.* (Natural Products Laboratory, University of North Carolina, Chapel Hill, NC, USA). Minimum Essential Medium (MEM), RPMI 1640 medium, fetal bovine serum (FBS), penicillin, and streptomycin were obtained from Gibco BRL Life Technologies (Grand Island, NY). EGTA, EDTA, leupeptin, dithiothreitol, phenylmethylsulfonyl fluoride (PMSF), propidium iodide (PI), dimethyl sulfoxide (DMSO), MTT (3-[4,5]-2,5-diphenyltetrazolium bromide), 4′-6-diamidino-2-phenylindole (DAPI), *N*-acetyl-_L_-cysteine (NAC) and etoposide were obtained from Sigma (St Louis, MO). Antibodies to various proteins were obtained from the following sources: anti-mouse and anti-rabbit IgGs, poly-ADP-ribosepolymerase (PARP), cyclin D1, cyclin E, cdk2, cdk4, p27, and TOP2β antibodies were purchased from Santa Cruz Biotechnology (Santa Cruz, CA); E2F-1, p21, p-Histone H2AX (Ser 139), p-ATM (Ser 1981), p-chk2 (Thr 68), p53 (Ser 15), p53 (Ser 20), cleaved caspase-3, caspase-9, and -8 were purchased from Cell Signaling Technology (Boston, MA); caspase-3 was purchased from Imgenex (San Diego, CA); p53, Retinoblastoma protein (Rb), TOP1 and TOP2α were purchased from BD Biosciences (San Diego, CA); actin was purchased from CHEMICON (Temecula, CA).

### Cell culture

Human cancer cell lines HCT 116, A549, ACHN, and A498 were purchased from the American Type Culture Collection (Manassas, VA). HCT 116, A549, and ACHN were cultured in RPMI 1640 medium and A498 cells were cultured in Minimum Essential Medium. Both media were supplemented with 10% FBS (v/v) and penicillin (100 U/mL)/streptomycin (100 µg/mL). Cultures were maintained in a humidified incubator at 37°C in 5% CO_2_/95% air.

### MTT assay

Cell viability was determined using MTT assay. In 96-well, 5,000 cells were seeded in complete media with presence or absence of NPRL-Z-1 and described in the previous study. Briefly, 100 µl MTT solution (0.5 mg/mL in phosphate-buffered saline; PBS) was added to each well. After 1 h incubation at 37°C, MTT solution was removed and DMSO was added to dissolve dye. Absorbance at 550 nm was measured using a microplate reader (Thermo Multiskan GO, Waltham, MA), using RPMI or MEN medium as a blank.

### *In situ* labeling of apoptotic cells

NPRL-Z-1-induced A498 cell apoptosis was detected using the terminal deoxynucleotidyl transferase-mediated nick-end labeling (TUNEL) staining assay. Briefly, cells were cultured in chamber slides for 24 h and treated with 10 µM NPRL-Z-1. After a 24 h treatment, A498 cells were washed twice with PBS and fixed for 10 min using ice-cold 1% paraformaldehyde. Staining was performed according to the TUNEL staining protocol provided by Promega Corporation (Madison, WI). Finally, photomicrographs of the TUNEL-stained cells were observed and photographed using Axioplan 2 fluorescence microscope (Carl Zeiss, Jena, Germany) equipped with a CCD camera (Nikon, Japan) at 20× magnification. Data were analyzed by AxioVision software.

### Cell death detection assay

NPRL-Z-1-induced apoptotic death was assessed using the Cell Death Detection ELISA^PLUS^ kit (Roche Diagnostics, Indianapolis, IN), which was designed for quantitative in vitro detection of mono- and oligonucleosomal DNA fragmentation. The manufacturer's protocol was applied from Roche and data were measured by microplate reader (Thermo Multiskan GO, Waltham, MA). Data were calculated and compared with those of a control group.

### Western blot analysis

Cells were lysed with lysis buffer as previously described and the samples were subjected to SDS-PAGE to detect protein phosphorylation, expression, and cleavage [26]. Briefly, proteins (30–60 µg) were separated by 10% polyacrylamide gel (Biored, Philadelphia, PA) and transferred to polyvinylidene fluoride membrane (Hoefer, Richmond, CA). Non-specific binding was blocked with 5% non-fat milk in PBS. The blots were probed with primary antibodies and incubated with horseradish peroxidase-conjugated goat anti-mouse or anti-rabbit antibodies. Finally, the membranes were visualized using an enhanced chemiluminescence kit (VISUAL PROTEIN, Taiwan).

### Flow cytometry analysis

Cells were seeded in six-well plates and treated with vehicle (0.1% DMSO) or the test compound at various concentrations for the indicated times, harvested by trypsinization, fixed with ice-cold 70% alcohol at −20°C overnight, centrifuged, and resuspended in 0.5 mL propidium iodide solution containing Triton X-100 (0.1%, v/v), RNase (100 µg/mL), and propidium iodide (80 µg/mL). DNA content was analyzed by fluorescence-activated cell sorting with the FACScan system and CellQuest software (Becton Dickinson, Mountain View, CA).

### Comet assay

Cells were seeded in 12-well plates and treated, collected, and resuspended in ice-cold PBS. Next, the resuspended cells were mixed with 1.5% low-melting point agarose and loaded onto a fully frosted slide precoated with 0.7% agarose. A coverslip was placed on the slide, which was then submerged in prechilled lysis solution (1% Triton X-100, 2.5 M NaCl, and 10 mM EDTA, pH 10.5) for 1 h at 4°C. After soaking in prechilled unwinding and electrophoresis buffer (0.3 M NaOH and 1 mM EDTA) for 20 min, the slides were subjected to electrophoresis for 15 min at 0.5 V/cm (20 mA), stained with 1× Sybr Gold (Molecular Probes), and nuclear images were visualized and captured using an Axioplan 2 fluorescence microscope (magnification, ×400; Carl Zeiss, Jena, Germany) equipped with a CCD camera (Optronics). More than hundreds of cells were scored to calculate the overall comet tail-positive cell percentage.

### Topoisomerase II relaxation assay

This assay was performed according to the manufacturer's protocol (TopoGEN, Port Orange, FL). Briefly, each reaction product was incubated at 37°C for 30 min and the reaction was stopped by adding stopping buffer. The products were analyzed by a 1% agarose gel in TAE buffer (40 mM Tris–acetate, pH 8.0, 1 mM EDTA) and stained with 0.5 µg/mL ethidium bromide for 10 min. After destaining with distilled water, the gel was photographed using a short-wavelength UV lamp (ChemiImager 5500; Alpha Innotech, Santa Clara, CA).

### Band depletion assay

Band depletion assay was performed to verify the presence of topoisomerase cleavage complexes that were retained on substrate DNA after topoisomerase poison treatment. Briefly, treated cells were lysed with SDS sample buffer (4% SDS/2%-β-mercaptoethanol, 20% glycerol, and 125 mM Tris HCl, pH 6.8), resolved by SDS-PAGE, and probed with anti-topoisomerase I/II antibodies.

### In vivo complex of enzyme (ICE) assay

After treatment with DMSO, NPRL-Z-1, or etoposide for 30 min, cells were collected and lysed with 1% sarkosyl in TE buffer (10 mM Tris–HCl pH 8.0 and 1 mM EDTA). The lysates were passed through a 26-gauge needle five times, placed on the top of a preformed cesium chloride step gradient (1.82, 1.72, 1.50, and 1.37 g/mL; volume, 2 mL each) in polyallomer tubes (Beckman Coulter, UK), and centrifuged at 20°C in a Beckman SW41 rotor at 31,000 rpm for 24 h. Next, 1 mL of each fraction was collected from the top of the tubes. A 100-µL aliquot of each fraction was diluted with equal volume of 25 mM sodium phosphate buffer (pH 6.5) and transferred to a polyvinylidene fluoride membrane, which was pre-soaked in sodium phosphate buffer for 15 min with a dot-blot apparatus (Biored, Philadelphia, PA). The membrane was immunoblotted with anti-TOP2 antibodies.

### Small interfering RNA transfection

Small interfering RNA (siRNA) against TOP2α, TOP2β, and the negative control were purchased from Ambion (Austin, TX), and the assay was performed as described previously [27]. Briefly, A498 cells were seeded in 6-cm dishes overnight, and then transfected with 10 µl negative control, TOP2α or TOP2β using 10 µl Lipofectamine 2000 (Invitrogen, Carlsbad, CA). After 24 h, the medium was replaced with growth medium and cells were treated with 2 µl DMSO or 10 µM NPRL-Z-1 for the indicated time.

### Measurement of reactive oxygen species

Intracellular reactive oxygen species level was detected using 2,7-dichlorofluoroscein diacetate (DCFH-DA). Cells were incubated in the absence or presence of the indicated agents for 0.5, 1 or 3 h. After DCFH-DA (10 µM) incubation for 30 min at 37°C, cells were harvested for the detection of ROS accumulation using FACScan flow cytometric analysis.

### Statistical analysis

All experiments were performed at least three times. Data expressed as mean ± SE for the indicated number of separate experiments. Statistical analysis of data was done with Student's *t* test. *P* values <0.05 were considered significant.

## Results

### Effect of NPRL-Z-1 on cell viability in human cancer cells

**Figure 1:**
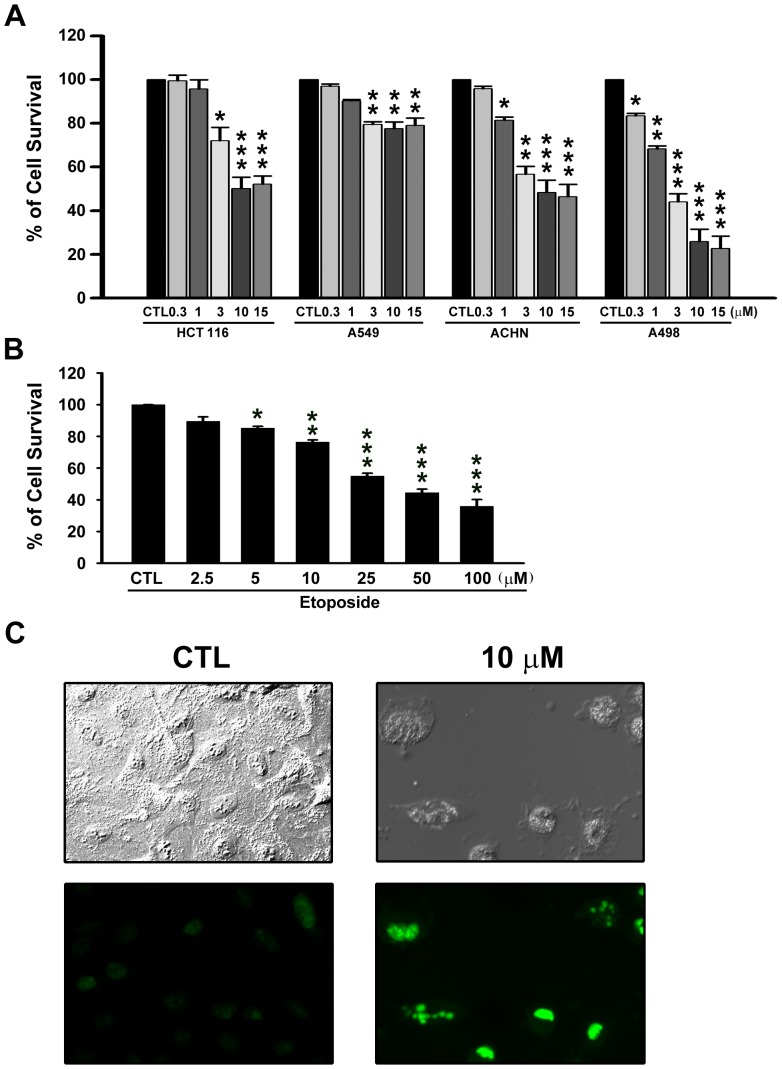
Effects of NPRL-Z-1 on cell viability in four human cancer cell lines. (A) HCT 116, A549, ACHN, and A498 were treated with NRRL-Z-1 at various concentrations for 48 h and analyzed using the MTT assay. (B) A498 cells were treated with vehicle or etoposide at various concentrations for 48 h and analyzed using the MTT assay. (C) Fluorescence microscopy of untreated or NPRL-Z-1-treated A498 cells for 24 h followed by TUNEL staining (at 20× magnification). Data are expressed as the mean percentage of control ± S.D. of three independent experiments. * *p*<0.05,** *p*<0.01, and *** *p*<0.001 compared with the control group.

Anticancer activity of NPRL-Z-1 was assessed in human colorectal cancer HCT 116 cells, human non-small cell lung carcinoma A549 cells, human renal carcinoma ACHN, and A498 cells. Cells were treated at various NPRL-Z-1 concentrations (0.3, 1, 3, 10, and 15 µM) for 48 h, and cell viability was determined using the MTT assay (Fig. 1A). NPRL-Z-1 inhibited cell viability in HCT116, ACHN, and A498 in a concentration-dependent manner, with IC_50_ values of 9.73 µM, 7.25 µM, and 2.38 µM, respectively. Therefore, the most potent cytotoxic effect of NPRL-Z-1 was observed in A498 cells. However, there was no significant decrease in cell viability among NPRL-Z-1-treated A549 cells even at a high concentration (15 µM). In addition, etoposide inhibited A498 cell viability in a concentration-dependent manner with an IC_50_ value of 39.52 µM (Fig. 1B). Moreover, NPRL-Z-1 significantly induced nuclear DNA fragmentation as determined by the TUNEL assay (Fig. 1C). In light field pictures, A498 cells appeared spindle-shaped, adhered to the surface of the culture plate and were growth confluent after 24 h incubation. After treatment with 10 µM NPRL-Z-1, prominent blebs on cell surface could be observed and the cells had started shrinking and rounding up, thus gradually detaching from the culture plate. Thus, these results demonstrated that NPRL-Z-1 induced cancer cell death and was 16-fold more potent than etoposide in A498 cells.

### NPRL-Z-1-induced cell apoptosis accompanied by downregulation of Akt signaling pathway in A498 cells

**Figure 2:**
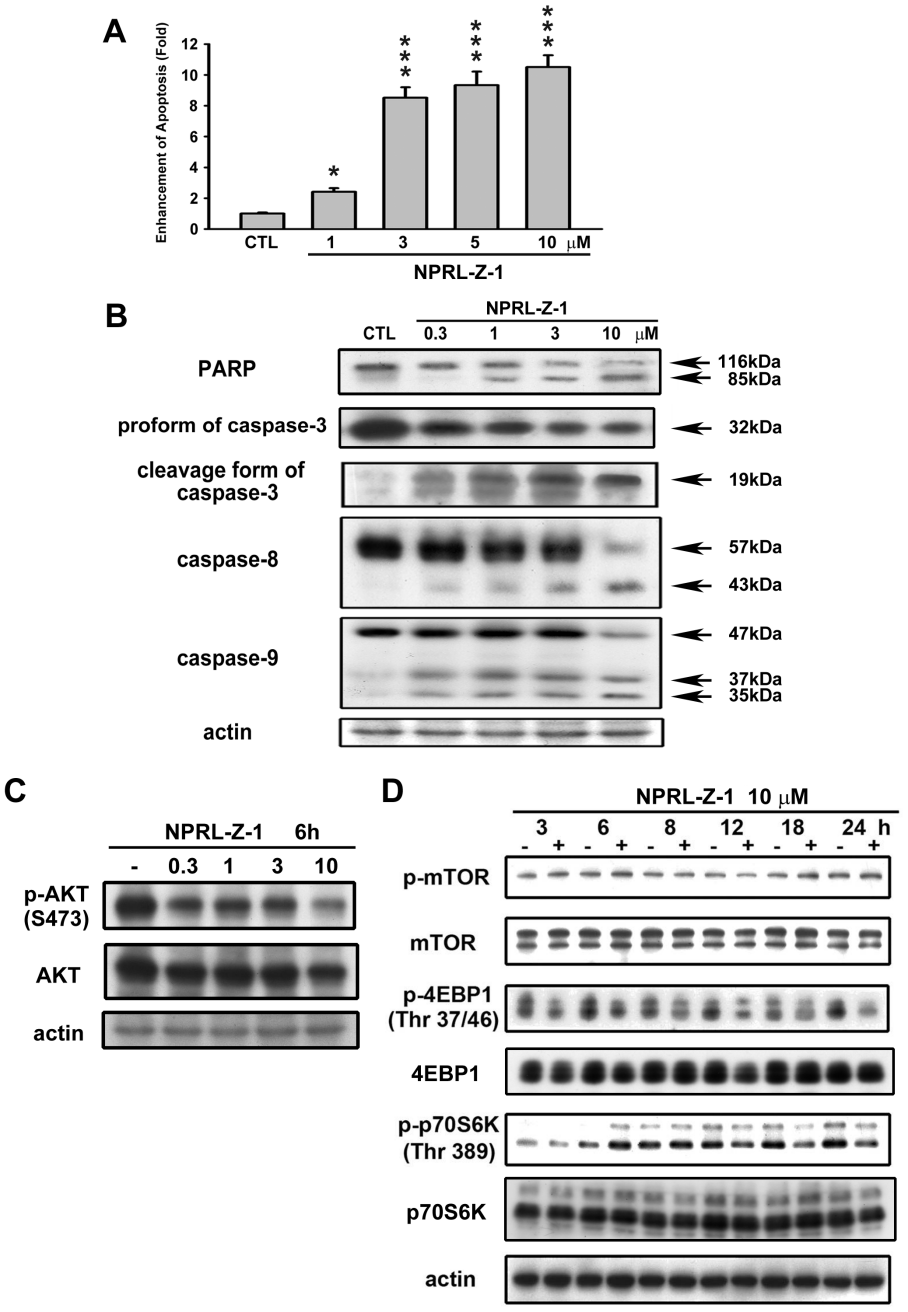
Effects of NPRL-Z-1 treatment on cell apoptosis induction and expression of apoptosis-related proteins in A498 cells. (A) Cells were treated with DMSO or NPRL-Z-1 at various concentrations (1, 3, 5, and 10 µM) for 24 h. Formation of cytoplasmic DNA was quantitatively measured by cell death ELISA^PLUS^ kit. Data are expressed as the mean percentage of control ± S.D. of three independent experiments. * *P*<0.05, and *** *P*<0.001 compared with the control group. A498 cells were incubated in the absence or presence of NPRL-Z-1 at various concentrations (0.3, 1, 3, and 10 µM) for 24 h (B) and 6 h (C), and cells were harvested and prepared for detection by Western blotting. (D) Cells were treated for indicated times, and the cell lysates were subjected to immunoblotting by using indicated antibodies.

The effects of NPRL-Z-1 on cell apoptosis induction were studied using a cell death detection ELISA kit. NPRL-Z-1 administration significantly triggered A498 cell apoptosis in a concentration-dependent manner (Fig. 2A). Upon apoptosis induction, the caspase cascade was also activated [28]. NPRL-Z-1 decreased pro-caspase-3, -8, and -9 expression and induced PARP and caspase-3, -8, and -9 cleavage (Fig. 2B). Furthermore, NPRL-Z-1 displayed a strong downregulatory effect on the Akt pathway. The phosphorylation of Akt, 4EBP1 and p70S6K were downregulated after NPRL-Z-1 treatment (Fig. 2C and 2D).

### Effect of NPRL-Z-1 on cell cycle progression

**Figure 3:**
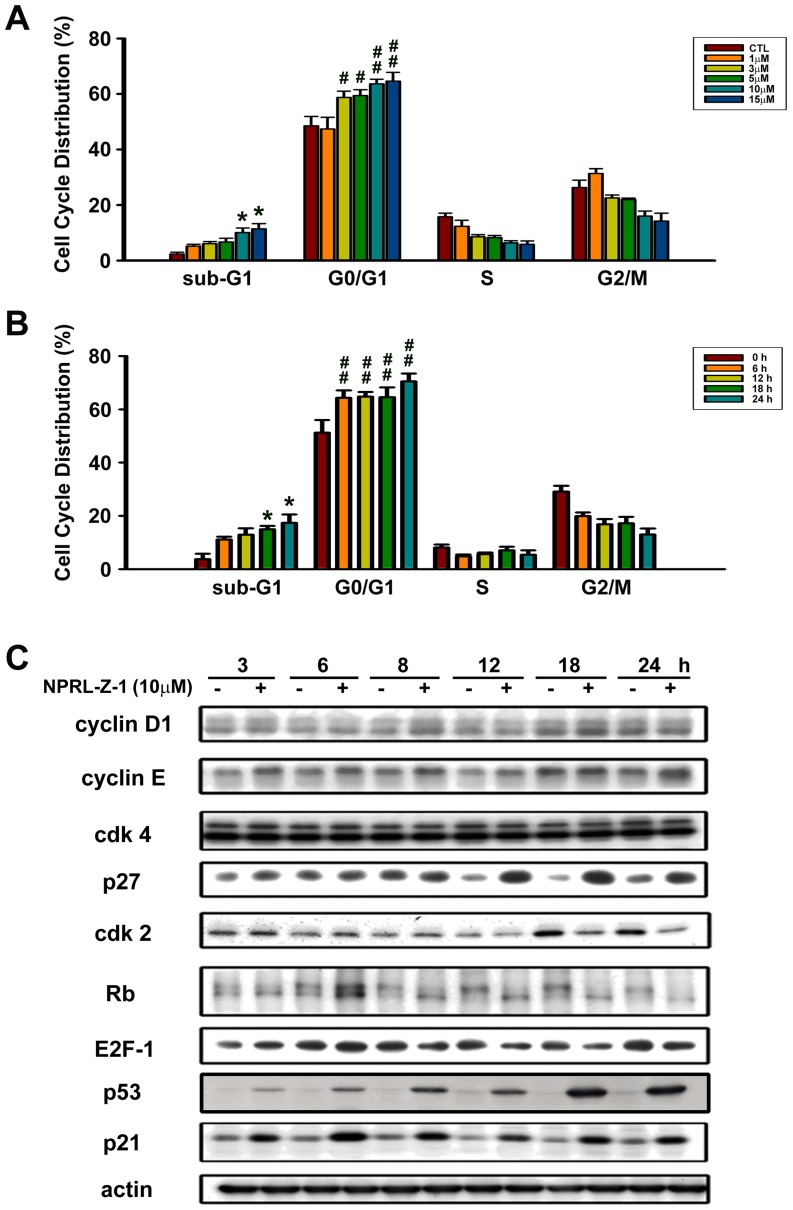
Effects of NPRL-Z-1 on cell cycle distribution and expression of cell cycle-related proteins in A498 cells. Cells were incubated with (A) DMSO or various concentrations of NPRL-Z-1 for 24 h and (B) DMSO or 10 µM NPRL-Z-1 for the indicated time periods. Cell cycle phase and cell apoptosis were determined by FACS as described in Materials and Methods. (C) A498 cells were incubated with DMSO or 10 µM NPRL-Z-1 for the indicated time periods. After treatment, cells were harvested and lysed for detection of the indicated proteins via western blotting. Data are expressed as the mean percentage of control ± S.D. of three independent experiments. *^, #^ *p*<0.05, and ^##^ *p*<0.01 compared with the control group.

Flow cytometry and propidium iodide staining were used to assess the effect of NPRL-Z-1 on cell cycle progression. After NPRL-Z-1 treatment, sub-G1 cell population increased in a concentration- and time-dependent manner (Fig. 3A and 3B). NPRL-Z-1 also induced cell cycle arrest in the G0/G1 phase among A498 cells.

Cell cycle progression is a complex process controlled by a subfamily of cyclin-dependent kinases (CDKs), which are modulated by several activators (cyclins) and inhibitors (Ink4 family and Cip/Kip family) [29]. To investigate the effects of NPRL-Z-1 on cell cycle regulatory molecules, expression levels of the indicated proteins was assessed by western blotting. The expression levels of p53, cyclin E and the CDK inhibitors, p21 and p27, upregulated after NPRL-Z-1 treatment (Fig. 3C). Moreover, NPRL-Z-1 induced Cdk2 upregulation during the first 8 h and downregulation during the last 6 h. However, Cdk4 and cyclin D1 expression levels remained unchanged. Retinoblastoma (Rb) tumor suppressor protein is a critical regulator of G1/S transition, which interacts with the E2F family of cell cycle transcription factors to repress gene transcription required for this transition [30]. The data from this study showed that NPRL-Z-1 decreased Rb phosphorylation and E2F-1 expression, which occurred within 8–24 h. Thus, these results suggested that NPRL-Z-1 induced G1 arrest in A498 cells.

### Effects of NPRL-Z-1 on DNA DSBs and DNA damage checkpoints

**Figure 4:**
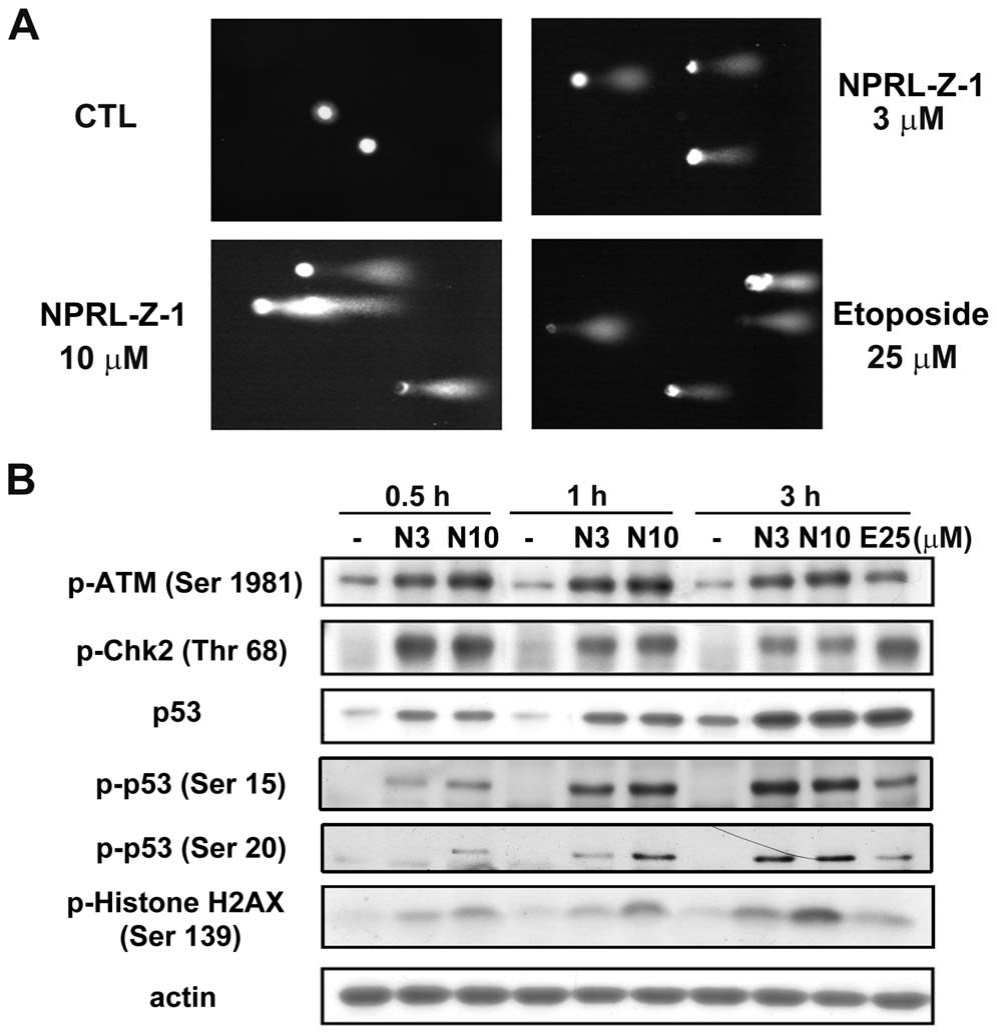
Effects of NPRL-Z-1 on DNA DSBs and DNA checkpoint pathway. (A) A498 cells were seeded and treated with NPRL-Z-1 or etoposide for 30 min and processed for the comet assay as detailed in Materials and Methods. (B) A498 cells were incubated with DMSO or 3 or 10 µM NPRL-Z-1 for the indicated time periods. After treatment, cells were harvested and lysed for detection of the expression of indicated protein via western blotting. N3, N10 and E25 indicated as NPRL-Z-1 3 µM, 10 µM and etoposide 25 µM, respectively.

The comet assay was performed to determine whether NPRL-Z-1 induced DNA damage. The results showed that NPRL-Z-1 induced chromosomal DNA strand breaks in A498 cells (Fig. 4A). ATM phosphorylation at serine (Ser) 1981 is an indicator of DNA damage [31]. A previous study indicated that DNA DSBs could induce histone H2AX phosphorylation at Ser 139 [32]. NPRL-Z-1 treatment of A498 cells increased the levels of p-ATM, p-Chk2, and p-histone H2AX (Fig. 4B). Moreover, p53 expression and phosphorylation (Ser 15 and 20) were upregulated. These results suggested that NPRL-Z-1 was a potent DNA-damaging agent that triggered activation of checkpoint signaling.

### Identification of TOP2 as a molecular target of NPRL-Z-1

**Figure 5:**
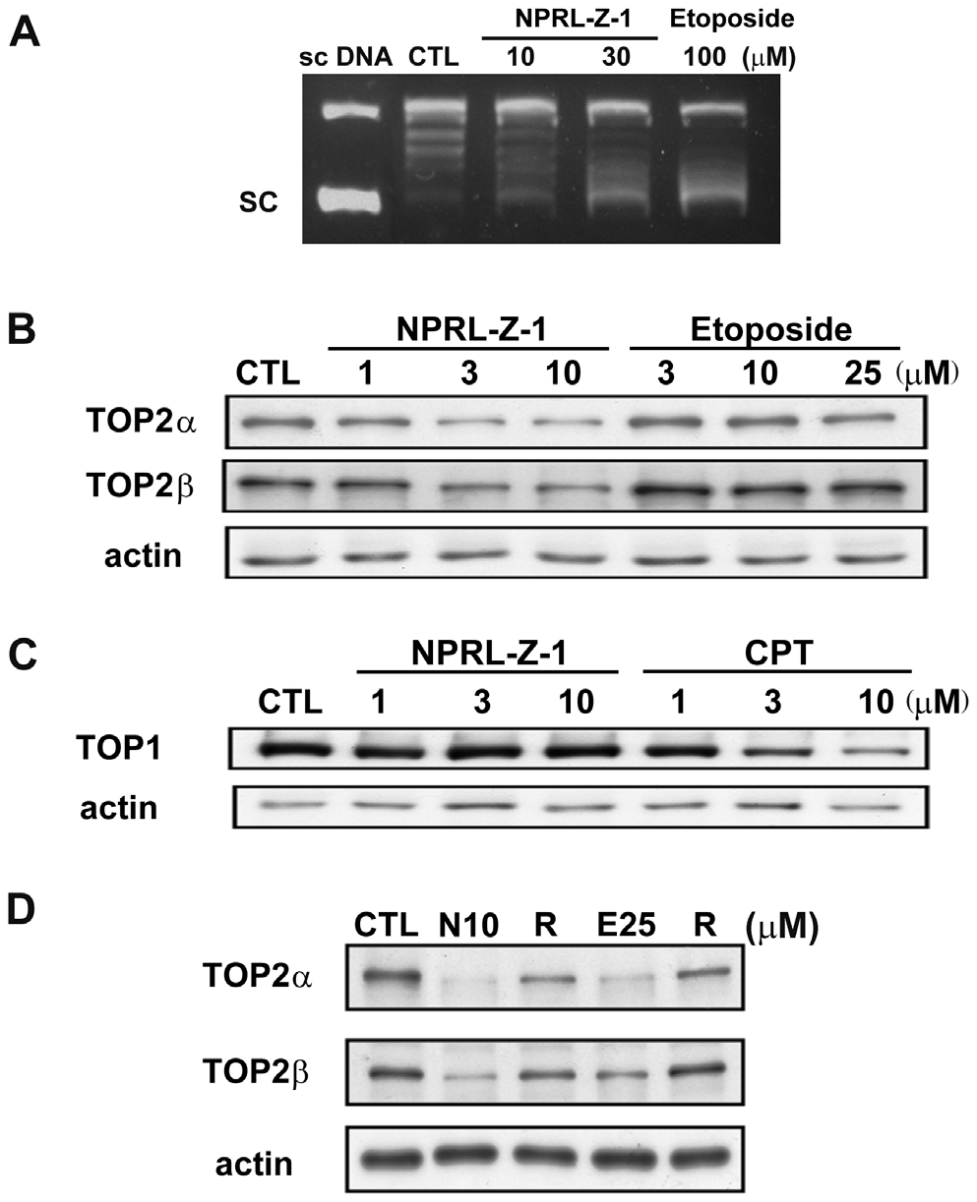
Effects of NPRL-Z-1 on TOP2 expression. (A) DNA relaxation assay. Lane 1: 0.3 pmol of negatively supercoiled DNA substrate and no protein; lane 2: DNA, TOP2, and DMSO; lanes 3–4: DNA, TOP2, and NPRL-Z-1; and lane 5: DNA, TOP2 and etoposide. (B) A498 cells were treated with NPRL-Z-1 or etoposide for 1 h to detect the depletion of free enzymes, TOP2α and TOP2β, using the band depletion assay. (C) A498 cells were treated with NPRL-Z-1 or camptothecin for 1 h to detect the depletion of free enzymes, TOP1, using the band depletion assay. (D) Restoration of depleted TOP2 expression. After treatment with NPRL-Z-1 or etoposide for 1 h, the medium was replaced with fresh growth medium and cells were incubated for another hour (R). Cells were then harvested and prepared for TOP2α and TOP2β detection via western blotting. N10 and E25 indicated as NPRL-Z-1 10 µM and etoposide 25 µM, respectively.

An *in vitro* DNA relaxation assay was performed to investigate whether TOP2 was involved in the NPRL-Z-1-induced DNA damage pathway. NPRL-Z-1 inhibited the ability of TOP2 to convert supercoiled DNA to relaxed DNA as well as etoposide (Fig. 5A). Furthermore, the band depletion assay was performed to detect free intracellular topoisomerases that were not covalently bound to cellular DNA. NPRL-Z-1 or etoposide treatment of A498 cells caused depletion of TOP2α and TOP2β, suggesting that NPRL-Z-1 increased intracellular accumulation of TOP2α- and TOP2β-mediated cleavable complexes (Fig. 5B). However, NPRL-Z-1 had no effect on TOP1–DNA complexes in A498 cells (Fig. 5C). The TOP1 poison camptothecin was used as a positive control. Moreover, after replacement of medium containing NPRL-Z-1 or etoposide with fresh growth medium, TOP2α and TOP2β expression was restored (Fig. 5D). These data clearly showed that NPRL-Z-1 dose-dependently stabilized TOP2 in A498 cells and the cleavable complexes were reversible.

### Effect of NPRL-Z-1 on formation of covalent TOP2–DNA complexes (TOP2cc)

**Figure 6:**
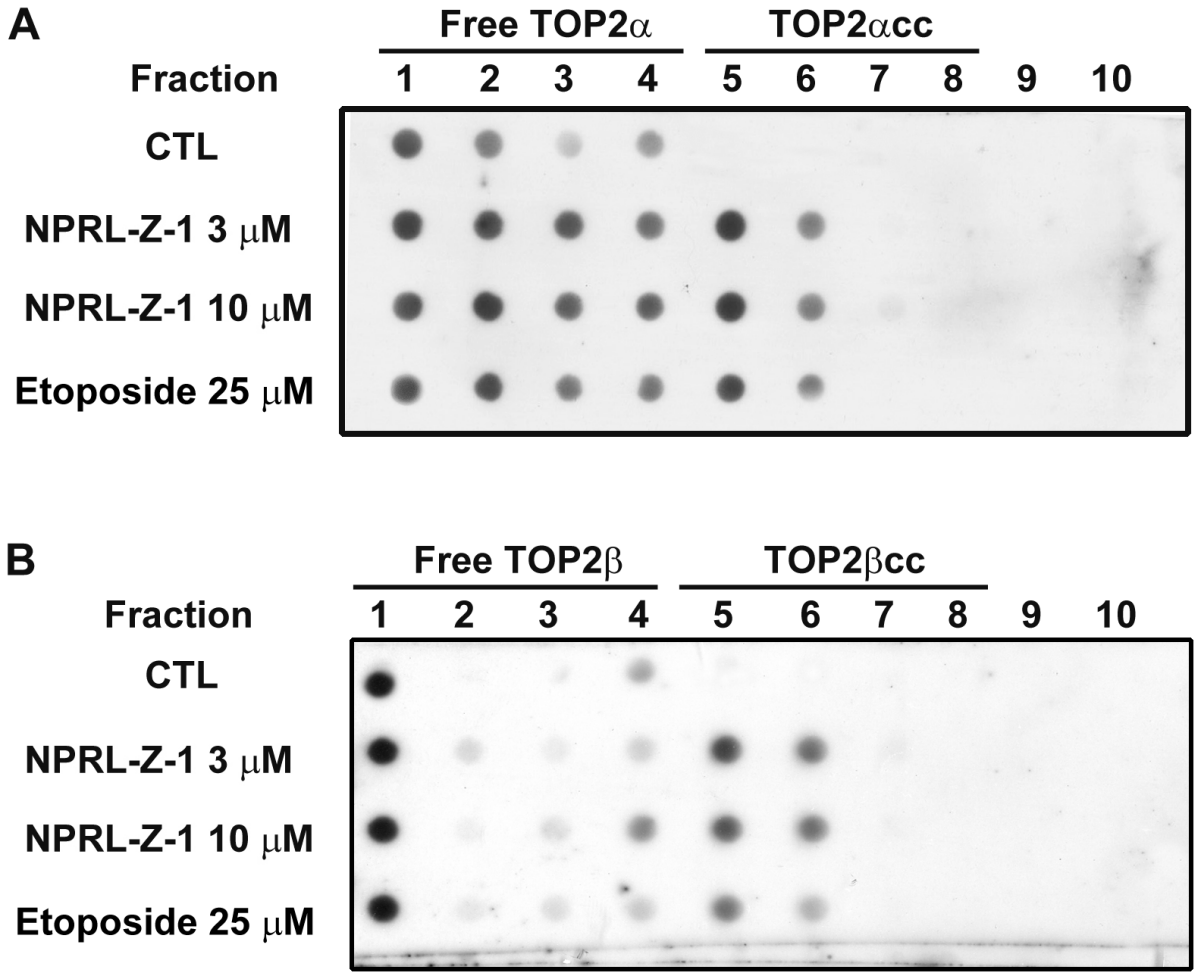
Effects of NPRL-Z-1 on TOP2cc formation in A498 cells. A498 cells were treated with NPRL-Z-1 or etoposide for 30 min and the ICE assay was performed to detect TOP2–DNA adduct formation as described in Materials and Methods. TOP2-free form was partitioned into fractions between 1 and 4. TOP2αcc (A) and TOP2βcc (B) were trapped between fractions 5 and 8, respectively.

Specific topoisomerase-genomic DNA cleavage complexes can be measured using ICE assay, in which complexes are separated from free TOP2 proteins by gradient centrifugation and detected with specific antibodies. Free TOP2 proteins were partitioned between fractions 1 and 4, and TOP2cc was trapped between fractions 5 and 8. The results showed that treatment with either NPRL-Z-1 or etoposide induced the formation of TOP2αcc (Fig. 6A) and TOP2βcc (Fig. 6B). Therefore, NPRL-Z-1 trapped TOP2 in the form of covalent protein–DNA complexes, demonstrating that NPRL-Z-1 is a novel TOP2 poison.

### TOP2 was required for NPRL-Z-1-induced cell apoptosis in A498 cells

**Figure 7:**
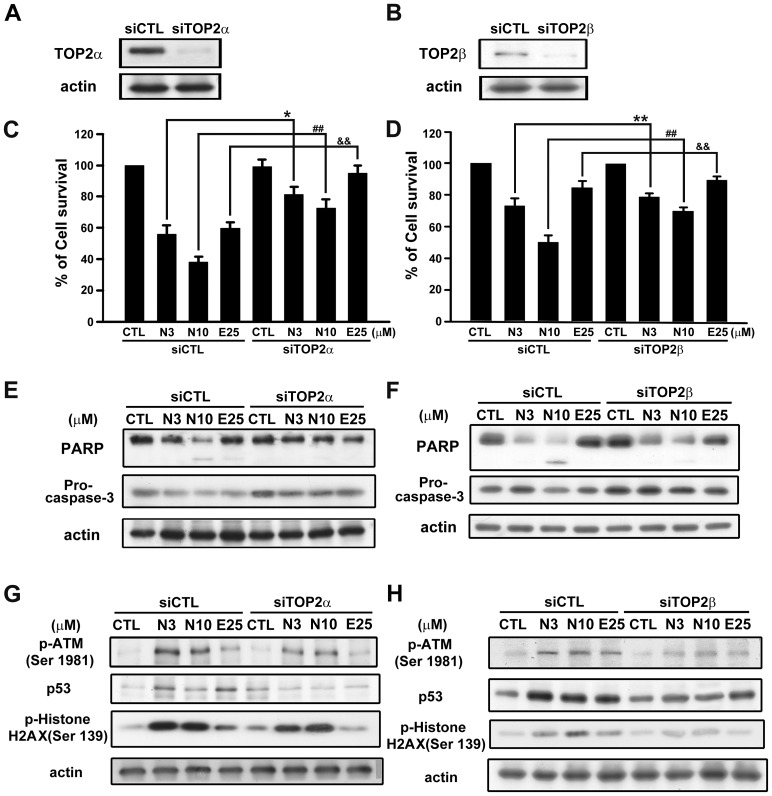
Effects of TOP2 in NPRL-Z-1-induced cell death and DNA damage signaling. TOP2α or TOP2β siRNA was transfected to evaluate expression of TOP2α (A) and TOP2β (B), cell viability using the MTT assay (C and D), expression of apoptosis-related proteins (PARP and pro-caspase-3) (E and F) for 24 h, and DNA damage-related proteins (p-ATM, p53, and p-histone H2AX) (G and H) for 1 h in A498 cells. Data are expressed as the mean percentage of control ± S.D. of three independent experiments. *^, &^ *p*<0.05, and **^, ##, &&^ *p*<0.01 compared with the treatment group. N3, N10 and E25 indicated as NPRL-Z-1 3 µM, 10 µM and etoposide 25 µM, respectively.

To determine the role of TOP2 in NPRL-Z-1-induced cell apoptosis, we transfected A498 cells with siRNA specifically targeted to TOP2α or TOP2β. TOP2α and TOP2β expression was downregulated in the transfected cells (Fig. 7A and 7B). Next, cell viability was measured using the MTT assay to assess the role of TOP2 in NPRL-Z-1-induced cell apoptosis. The results showed that TOP2α or TOP2β knockdown protected A498 cells from NPRL-Z-1-induced cell death (Fig. 7C and 7D) and was also involved in PARP cleavage and pro-caspase-3 downregulation (Fig. 7E and 7F). Furthermore, NPRL-Z-1-induced DNA damage checkpoints, such as p-ATM, p53, and p-histone H2AX, reduced after TOP2α or TOP2β silencing (Fig. 7G and 7H). These data indicated that TOP2 played a crucial role in NPRL-Z-1-induced activation of DNA damage signaling and the apoptotic pathway.

### NPRL-Z-1-induced Cell Apoptosis through Reactive Oxygen Species Generation

**Figure 8:**
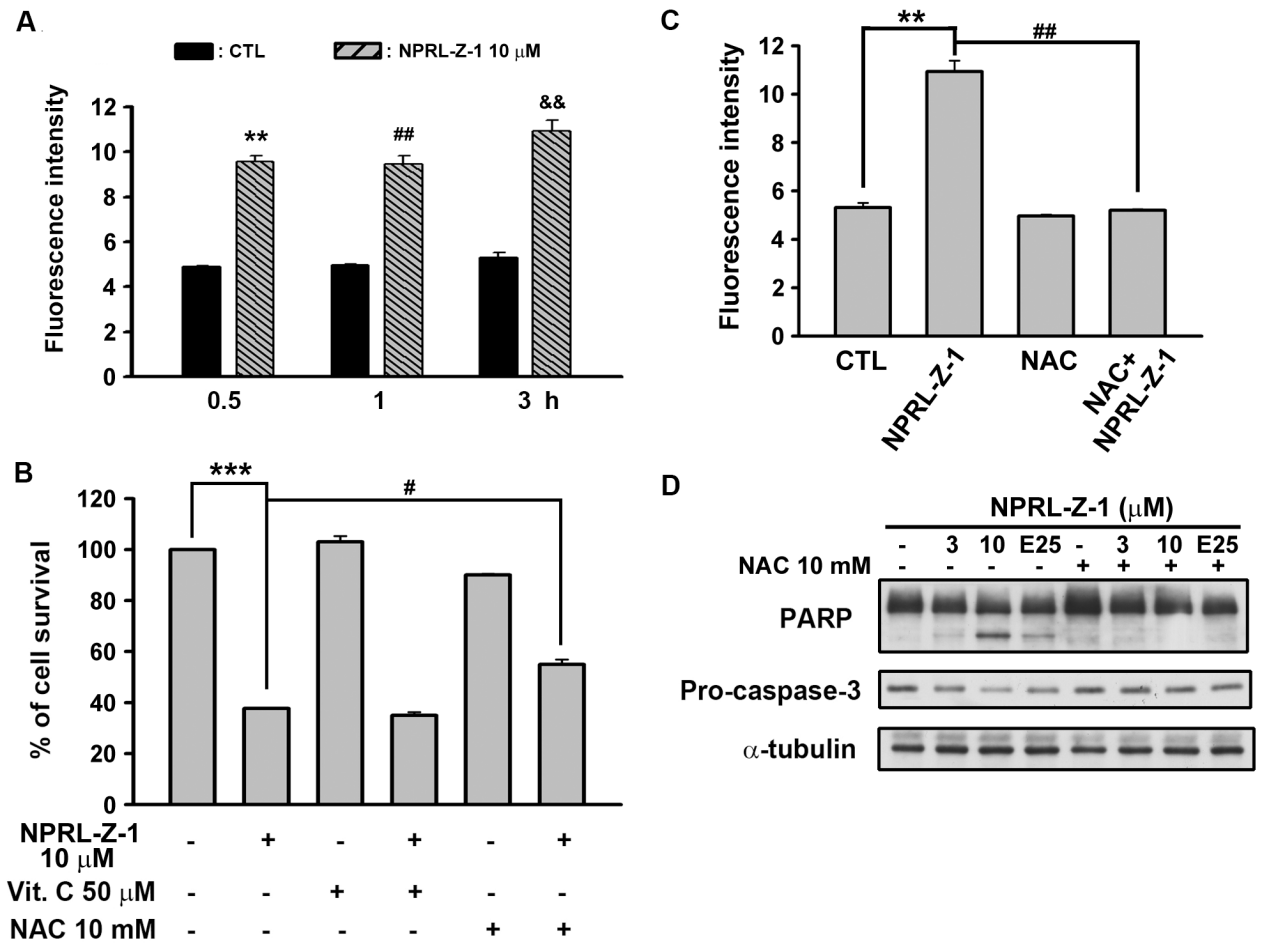
Effect of NPRL-Z-1 on cellular ROS accumulation in A498 cells. A498 cells were incubated in the absence or presence of NPRL-Z-1 (10 µM) for indicated time. The fluorescent intensity of DCFH-DA was detected by flow cytometric analysis. ** *P*<0.01 compared with the 0.5 h-time point control group. ^##^ *P*<0.05 compared with the 1 h-time point control group. ^&&^ *P*<0.01 compared with the 3 hr-time point control group. (B) Different ROS scavengers were preincubated for 30 min and cell viability was determined by MTT assay. *** *P*<0.001 compared with the control group. ^#^ *P*<0.05 compared with the NPRL-Z-1-treated group. Treatment of NAC inhibited NPRL-Z-1-induced ROS generation (C, ** *P*<0.01 and ^##^ *P*<0.01 compared with the NPRL-Z-1-treated group.). (D) NAC (10 mM) reduced the apoptosis-related results in NPRL-Z-1-treated cells for 24 h. E25 indicated as etoposide 25 µM.

It has been reported that ROS is involved in cancer cell apoptosis induced by chemotherapeutic agents and radiotherapy [33]. Therefore, we detected the intracellular ROS level by using flow cytometry. As shown in Fig. 8A, a significant generation of ROS was observed after treatment with NPRL-Z-1. Next, two ROS scavengers (vitamin C and NAC) were studied to determine which antioxidant could rescue the cells from NPRL-Z-1-induced cell death. Notably, only NAC could protected from NPRL-Z-1-induced cell death (Fig. 8B) and blocked NPRL-Z-1-induced ROS generation (Fig. 8C). In addition, the downstream signals, caspase-3 and PARP were restored by NAC (Fig. 8D). These results suggested that the cell death induced by NPRL-Z-1, in the A498 cells, was also mediated by the accumulation of ROS pathway.

## Discussion

RCC is the most lethal cancer among urological tumors and there are no effective biomarkers in the prognostic or predictive assessment till present day. In addition, approximately 30% of all RCC patients develop metastatic lesions [34]. Because metastatic RCC is resistant to radiation therapy and chemotherapy, novel therapeutic treatment strategies and finding effective biomarkers are needed urgently. Parker *et al.* discovered that higher expression of TOP2α is associated with an increased risk of cancer-related death following surgery for clinically localized clear cell renal cell carcinoma [35]. Those tumors classified as TOP 2α high had more aggressive pathologic features. It provides a good reason to design the TOP 2-target therapy in RCC.

At the core of the DNA damage signaling apparatus is a pair of related protein kinases ATM and ATR (ATM and Rad3-related) that are activated by DNA damage. ATM senses DSBs, whereas ATR senses ssDNA generated by processing DSBs, as well as ssDNA present at stalled replication forks [36]. Both activated kinases then phosphorylate and activate several downstream effectors to arrest the cell cycle for DNA repair, including Chk1 and Chk2 (checkpoint kinases), which initiate a secondary wave of phosphorylation events to extend signaling. Here, the data showed that NPRL-Z-1induced DNA DSBs and increased phosphorylation of ATM and Chk2. Furthermore, ATM was reportedly involved in regulating multiple cell cycle checkpoints (G1/S, S, and G2/M phases) after DNA damage [37]. NPRL-Z-1 treatment induced ATM phosphorylation at Ser 1981, which has been shown to regulate ATM activity and p53 phosphorylation at Ser 15. In addition, DSBs triggered Chk2 phosphorylation at Thr 68 [38]. Activated Chk2 subsequently phosphorylated p53 at Ser 20 [39], which upregulated the expression of DNA-damage and cell cycle arrest-related genes (p21 and p27) [40]. p21, a potent Cdk inhibitor bound and inactivated cyclin E and the Cdk2 complex, led to G1 cell cycle arrest through inhibition of Rb phosphorylation [41]. In addition, histone H2AX was required for p21-induced cell cycle arrest after replication stalling [42]. The results of this study showed that NPRL-Z-1 activated the ATM signaling pathway and stimulated p53 accumulation, subsequently upregulated the cell cycle regulators p21, p27 and cyclin E to cause G1 cell cycle arrest.

Many effective anticancer drugs target topoisomerases, including etoposide. Previous research has demonstrated that etoposide inhibited TOP2 activity by stabilizing a covalent TOP2–DNA complex [12]. However, etoposide exhibits serious side effects, drug-resistance, and poor water solubility, limiting its use. Alternatively, NPRL-Z-1 was designed to enhance DNA TOP2 inhibition, overcome drug resistance, and increase water solubility of etoposide analogues by extending the bulky substituent at C7. The data from the present study revealed that NPRL-Z-1 inhibited the unwinding activity of TOP2, but not topoisomerase I, by supercoiling of DNA, which likely led to replication inhibition of A498 cells. Moreover, the relationship between TOP2 expression and drug sensitivity in tumor cells has been described [43], [44]. As shown in Fig. 1A, cytotoxicity was most potent in A498 cells which may resulted from TOP2α and β expression levels were higher than other cancer cell lines (Fig. S1 in File S1). Furthermore, NPRL-Z-1 induced G1-phase arrest but etoposide induced G2/M-phase arrest in A498 cells (Fig. S2 in File S1). Jin *et al.* had found that tumor cells arrested in the G0/G1-phase of the cell cycle are more susceptible to TRAIL-induced apoptosis compared to cells arrested in other cell cycle phases [45]. They found that caspase-9 inhibition can partially block the enhanced TRAIL-induced apoptosis in G0/G1-arrested SW480 cells, but not apoptosis in unsynchronized cells. These results suggested that the mitochondrial pathway is more likely to be activated during apoptosis when cells are arrested in G0/G1. Therefore, it suggested that NRPL-Z-1 arrested cell cycle in G1 phase would help for increasing drug sensitivity. In addition, we also found that NPRL-Z-1 induced similar mechanism in ACHN cell (Fig. S3 in File S1). Moreover, NPRL-Z-1 was 16-fold more potent than etoposide in A498 and ACHN cells and may only 5-fold in A549 cell, suggesting NPRL-Z-1 had better efficacy than etoposide and in renal carcinoma cell lines.

Many clinically useful anticancer drugs, including etoposide, *m*-AMSA, doxorubicin, and mitoxantrone, act through a common cytotoxic mechanism by inducing TOP2cc formation [18]. Etoposide induces TOP2cc formation and activates many molecules in response to DNA damage, such as ATM, histone H2AX, Chk1/2, p53, and replication protein A (RPA) [46]-[51], which subsequently induce various cellular responses, including cell cycle arrest, non-homologous end joining, homologous recombination repair, and cell apoptosis [47], [52]–[54]. Our results revealed that NPRL-Z-1 induced TOP2cc formation in A498 cells which indicated NPRL-Z-1 acted as a TOP2 poison. To validate whether TOP2 was required for NPRL-Z-1-induced cell death, we examined the effect of siRNA-mediated knockdown of TOP2α and β. The results indicated that silencing of TOP2α and β reversed NPRL-Z-1-induced cell apoptosis and DNA damage signaling.

Previous reports had indicated that chemotherapeutic agents may be selectively toxic to tumor cells because of increasing oxidant stress due to excess of ROS [33], [55], [56]. In our study, NPRL-Z-1 was shown to induce ROS accumulation and significantly reversed by NAC, a precursor of glutathione (GSH). In addition, NAC restored the cell viability and downregulation of pro-caspase-3 and PARP cleavage after NPRL-Z-1 treatment in A498 cells. It has been reported that GSH depletion is associated with ROS generation and apoptosis induction [57], [58]. Previous studies have shown that etoposide could cause GSH depletion [59], [60]. Therefore, it suggested that ROS played a important role in NPRL-Z-1-induced cell apoptosis pathway.

The PI3K/Akt signaling pathway plays a crucial role in regulating cell proliferation, growth, apoptosis, survival, and metabolism via phosphorylation of a variety of substrates, and inhibition of Akt phosphorylation has been suggested as a novel target for therapeutic agents in human cancer [61]. Sourbier *et al.* showed that this pathway is constitutively activated in human RCC and plays an essential role in RCC progression through the inhibition of tumor cell apoptosis [62]. Horiguchi *et al.* [63] evaluated Akt activation by immunohistochemistry in 48 human renal cell carcinoma biopsies and investigated its association with pathologic features and clinical outcome. They found that elevated Akt activation could be a common finding, especially in high-grade tumors and metastatic disease, and thus suggested that Akt might have an important role in the pathogenesis and progression of renal cell carcinoma. Here NPRL-Z-1 treatment markedly suppressed the activation of Akt and related downstream proteins. Therefore, we expected NPRL-Z-1 may be capable of preventing tumor resistance and needed for further investigation.

In conclusion, NPRL-Z-1 induced DNA DSBs, TOP2cc formation, and ROS production in A498 cells. When ATM was activated by DSBs, p53 and p21 expression increased and cell cycle was arrested. Ultimately, NPRL-Z-1 induced cell apoptosis. Thus, these results provide compelling evidence that there is potential for NPRL-Z-1 to be developed as a promising anticancer drug.

## Supporting Information

File S1 **Supporting Figures.** **Figure S1. Expression of TOP2α or TOP2β in A498, ACHN, and A549 cells.** Three human cancer cell lines (A498, ACHN, and A549) were seeded overnight, harvested, and prepared for detection of TOP2α or TOP2β expression via western blotting. **Figure S2. Effects of etoposide on cell cycle distribution in A498 cells.** Cells were incubated with vehicle or various concentrations of etoposide for 24 h and detected cell cycle distribution by flow cytometry. **Figure S3. Effects of NPRL-Z-1 in ACNH cells.** (A) NPRL-Z-1 induced PARP cleavage. (B) NPRL-Z-1 induced DNA checkpoints activation. (C) Transfection of siTOP2α or siTOP2β could reverse NPRL-Z-1-induced cell death in ACHN cells.
(DOCX)

## References

[1] Hurley LH (2002) DNA and its associated processes as targets for cancer therapy. Nat Rev Cancer 2: 188–200. 11990855 10.1038/nrc749

[2] Nitiss JL (2009) Targeting DNA topoisomerase II in cancer chemotherapy. Nat Rev Cancer 9: 338–350. 19377506 10.1038/nrc2607 PMC2748742

[3] Wang H-K, Morris-Natschke SL, Lee K-H (1997) Recent advances in the discovery and development of topoisomerase inhibitors as antitumor agents. Medicinal Research Reviews 17: 367–425. 9211397 10.1002/(sici)1098-1128(199707)17:4<367::aid-med3>3.0.co;2-u

[4] Haglof KJ, Popa E, Hochster HS (2006) Recent developments in the clinical activity of topoisomerase-1 inhibitors. Update on Cancer Therapeutics 1: 117–145. 16110608

[5] Pommier Y (2009) DNA Topoisomerase I Inhibitors: Chemistry, Biology, and Interfacial Inhibition. Chemical Reviews 109: 2894–2902. 19476377 10.1021/cr900097c PMC2707511

[6] Hande KR (1998) Clinical applications of anticancer drugs targeted to topoisomerase II. Biochimica et Biophysica Acta (BBA) - Gene Structure and Expression 1400: 173–184. 9748560 10.1016/s0167-4781(98)00134-1

[7] Hande KR (1998) Etoposide: four decades of development of a topoisomerase II inhibitor. European Journal of Cancer 34: 1514–1521. 9893622 10.1016/s0959-8049(98)00228-7

[8] Champoux JJ (2001) DNA TOPOISOMERASES: Structure, Function, and Mechanism. Annual Review of Biochemistry 70: 369–413. 10.1146/annurev.biochem.70.1.369 11395412

[9] Goto T, Wang JC (1982) Yeast DNA topoisomerase II. An ATP-dependent type II topoisomerase that catalyzes the catenation, decatenation, unknotting, and relaxation of double-stranded DNA rings. Journal of Biological Chemistry 257: 5866–5872. 6279616

[10] Wang JC (2002) Cellular roles of dna topoisomerases: a molecular perspective. Nature Reviews Molecular Cell Biology 3: 430–440. 12042765 10.1038/nrm831

[11] Nitiss JL (2009) DNA topoisomerase II and its growing repertoire of biological functions. Nature Reviews Cancer 9: 327–337. 19377505 10.1038/nrc2608 PMC2730144

[12] Deweese JE, Osheroff N (2009) The DNA cleavage reaction of topoisomerase II: wolf in sheep's clothing. Nucleic Acids Research 37: 738–748. 19042970 10.1093/nar/gkn937 PMC2647315

[13] Baxter J, Diffley JFX (2008) Topoisomerase II Inactivation Prevents the Completion of DNA Replication in Budding Yeast. Molecular Cell 30: 790–802. 18570880 10.1016/j.molcel.2008.04.019

[14] Stewart L, Redinbo MR, Qiu X, Hol WGJ, Champoux JJ (1998) A Model for the Mechanism of Human Topoisomerase I. Science 279: 1534–1541. 9488652 10.1126/science.279.5356.1534

[15] Brown P, Cozzarelli N (1979) A sign inversion mechanism for enzymatic supercoiling of DNA. Science 206: 1081–1083. 227059 10.1126/science.227059

[16] Woessner R, Mattern M, Mirabelli C, Johnson R, Drake F (1991) Proliferation- and cell cycle-dependent differences in expression of the 170 kilodalton and 180 kilodalton forms of topoisomerase II in NIH-3T3 cells. Cell Growth Differ 2: 209–214. 1651102

[17] Hande KR (2008) Topoisomerase II inhibitors. Update on Cancer Therapeutics 3: 13–26.

[18] Li T-K, Liu LF (2001) Tumor cell death induced by topoisomerase-targeting drugs. Annual Review of Pharmacology and Toxicology 41: 53–77. 10.1146/annurev.pharmtox.41.1.53 11264450

[19] Chikamori K, Grozav AG, Kozuki T, Grabowski D, Ganapathi R, et al (2010) DNA Topoisomerase II Enzymes as Molecular Targets for Cancer Chemotherapy. Current Cancer Drug Targets 10: 758–771. 20578986 10.2174/156800910793605785

[20] Felix CA, Kolaris CP, Osheroff N (2006) Topoisomerase II and the etiology of chromosomal translocations. DNA Repair 5: 1093–1108. 16857431 10.1016/j.dnarep.2006.05.031

[21] McClendon AK, Osheroff N (2007) DNA topoisomerase II, genotoxicity, and cancer. Mutation Research/Fundamental and Molecular Mechanisms of Mutagenesis 623: 83–97. 17681352 10.1016/j.mrfmmm.2007.06.009 PMC2679583

[22] Hawtin RE, Stockett DE, Byl JAW, McDowell RS, Tan N, et al (2010) Voreloxin Is an Anticancer Quinolone Derivative that Intercalates DNA and Poisons Topoisomerase II. PLoS ONE 5: e10186. 20419121 10.1371/journal.pone.0010186 PMC2855444

[23] Guo A, Marinaro W, Hu P, Sinko PJ (2002) Delineating the Contribution of Secretory Transporters in the Efflux of Etoposide Using Madin-Darby Canine Kidney (MDCK) Cells Overexpressing P-Glycoprotein (Pgp), Multidrug Resistance-Associated Protein (MRP1), and Canalicular Multispecific Organic Anion Transporter (cMOAT). Drug Metabolism and Disposition 30: 457–463. 11901101 10.1124/dmd.30.4.457

[24] Winick NJ, McKenna RW, Shuster JJ, Schneider NR, Borowitz MJ, et al (1993) Secondary acute myeloid leukemia in children with acute lymphoblastic leukemia treated with etoposide. Journal of Clinical Oncology 11: 209–217. 8426196 10.1200/JCO.1993.11.2.209

[25] Xiao Z, Bastow KF, Vance JR, Sidwell RS, Wang H-K, et al (2004) Antitumor Agents. 234.† Design, Synthesis, and Biological Evaluation of Novel 4β-[(4″-Benzamido)-Amino]-4′-O-Demethyl-Epipodophyllotoxin Derivatives. Journal of Medicinal Chemistry 47: 5140–5148. 15456257 10.1021/jm030609l

[26] Wu S-Y, Leu Y-L, Chang Y-L, Wu T-S, Kuo P-C, et al (2012) Physalin F Induces Cell Apoptosis in Human Renal Carcinoma Cells by Targeting NF-kappaB and Generating Reactive Oxygen Species. PLoS ONE 7: e40727. 22815798 10.1371/journal.pone.0040727 PMC3398016

[27] Hong Y, Sang M, Shang C, Xue Y-X, Liu Y-H (2012) Quantitative analysis of topoisomerase II alpha and evaluation of its effects on cell proliferation and apoptosis in glioblastoma cancer stem cells. Neuroscience Letters 518: 138–143. 22569122 10.1016/j.neulet.2012.04.071

[28] Boatright KM, Salvesen GS (2003) Mechanisms of caspase activation. Current Opinion in Cell Biology 15: 725–731. 14644197 10.1016/j.ceb.2003.10.009

[29] Malumbres M, Barbacid M (2009) Cell cycle, CDKs and cancer: a changing paradigm. Nat Rev Cancer 9: 153–166. 19238148 10.1038/nrc2602

[30] Harbour JW, Dean DC (2000) Rb function in cell-cycle regulation and apoptosis. Nat Cell Biol 2: E65–E67. 10783254 10.1038/35008695

[31] Kurz EU, Lees-Miller SP (2004) DNA damage-induced activation of ATM and ATM-dependent signaling pathways. DNA Repair 3: 889–900. 15279774 10.1016/j.dnarep.2004.03.029

[32] Rogakou EP, Pilch DR, Orr AH, Ivanova VS, Bonner WM (1998) DNA Double-stranded Breaks Induce Histone H2AX Phosphorylation on Serine 139. Journal of Biological Chemistry 273: 5858–5868. 9488723 10.1074/jbc.273.10.5858

[33] Pelicano H, Carney D, Huang P (2004) ROS stress in cancer cells and therapeutic implications. Drug Resistance Updates 7: 97–110. 15158766 10.1016/j.drup.2004.01.004

[34] van der Veldt AA, Haanen JB, van den Eertwegh AJ, Boven E (2010) Targeted therapy for renal cell cancer: current perspectives. Discov Med 10: 394–405. 21122471

[35] Parker AS, Eckel-Passow JE, Serie D, Hilton T, Parasramka M, et al.. (2013) Higher Expression of Topoisomerase II Alpha Is an Independent Marker of Increased Risk of Cancer-specific Death in Patients with Clear Cell Renal Cell Carcinoma. European Urology. 10.1016/j.eururo.2013.12.017 PMC4071134 24388441

[36] Matsuoka S, Ballif BA, Smogorzewska A, McDonald ER III, Hurov KE, et al (2007) ATM and ATR Substrate Analysis Reveals Extensive Protein Networks Responsive to DNA Damage. Science 316: 1160–1166. 17525332 10.1126/science.1140321

[37] Goodarzi AA, Block WD, Lees-Miller SP (2003) The role of ATM and ATR in DNA damage-induced cell cycle control. Progress in cell cycle research 5: 393–411. 14593734

[38] Matsuoka S, Huang M, Elledge SJ (1998) Linkage of ATM to Cell Cycle Regulation by the Chk2 Protein Kinase. Science 282: 1893–1897. 9836640 10.1126/science.282.5395.1893

[39] Delia D, Fontanella E, Ferrario C, Chessa L, Mizutani S (2003) DNA damage-induced cell-cycle phase regulation of p53 and p21waf1 in normal and ATM-defective cells. Oncogene 22: 7866–7869. 14586414 10.1038/sj.onc.1207086

[40] Dasika GK, Lin SC, Zhao S, Sung P, Tomkinson A, et al (1999) DNA damage-induced cell cycle checkpoints and DNA strand break repair in development and tumorigenesis. Oncogene 18: 7883–7899. 10630641 10.1038/sj.onc.1203283

[41] Costanzi-Strauss E, Strauss BE, Naviaux RK, Haas M (1998) Restoration of Growth Arrest by p16INK4, p21WAF1, pRB, and p53 Is Dependent on the Integrity of the Endogenous Cell-Cycle Control Pathways in Human Glioblastoma Cell Lines. Experimental Cell Research 238: 51–62. 9457056 10.1006/excr.1997.3810

[42] Fragkos M, Jurvansuu J, Beard P (2009) H2AX Is Required for Cell Cycle Arrest via the p53/p21 Pathway. Molecular and Cellular Biology 29: 2828–2840. 19273588 10.1128/MCB.01830-08 PMC2682023

[43] Son YS, Suh JM, Ahn SH, Kim JC, Yi JY, et al (1998) Reduced activity of topoisomerase II in an Adriamycin-resistant human stomach-adenocarcinoma cell line. Cancer Chemother Pharmacol 41: 353–360. 9523730 10.1007/s002800050751

[44] Morgan SE, Cadena RS, Raimondi SC, Beck WT (2000) Selection of Human Leukemic CEM Cells for Resistance to the DNA Topoisomerase II Catalytic Inhibitor ICRF-187 Results in Increased Levels of Topoisomerase IIα and Altered G2/M Checkpoint and Apoptotic Responses. Molecular Pharmacology 57: 296–307. 10648639

[45] Jin Z, Dicker DT, El-Deiry WS (2002) Enhanced Sensitivity of G1 Arrested Human Cancer Cells Suggests a Novel Therapeutic Strategy Using a Combination of Simvastatin and TRAIL. Cell Cycle 1: 79–86. 12429913

[46] Ye R, Bodero A, Zhou B-B, Khanna KK, Lavin MF, et al (2001) The Plant Isoflavenoid Genistein Activates p53 and Chk2 in an ATM-dependent Manner. Journal of Biological Chemistry 276: 4828–4833. 11096068 10.1074/jbc.M004894200

[47] Sordet O, Khan QA, Kohn KW, Pommier Y (2003) Apoptosis Induced by Topoisomerase Inhibitors. Current Medicinal Chemistry - Anti-Cancer Agents 3: 271–290. 12769773 10.2174/1568011033482378

[48] Banáth JP, Olive PL (2003) Expression of phosphorylated histone H2AX as a surrogate of cell killing by drugs that create DNA double-strand breaks. Cancer Research 63: 4347–4350. 12907603

[49] Zhao H, Piwnica-Worms H (2001) ATR-Mediated Checkpoint Pathways Regulate Phosphorylation and Activation of Human Chk1. Molecular and Cellular Biology 21: 4129–4139. 11390642 10.1128/MCB.21.13.4129-4139.2001 PMC87074

[50] Shao R-G, Cao C-X, Zhang H, Kohn KW, Wold MS, et al (1999) Replication-mediated DNA damage by camptothecin induces phosphorylation of RPA by DNA-dependent protein kinase and dissociates RPA:DNA-PK complexes. EMBO J 18: 1397–1406. 10064605 10.1093/emboj/18.5.1397 PMC1171229

[51] Piret B, Piette J (1996) Topoisomerase Poisons Activate the Transcription Factor NF-κB in ACH-2 and CEM Cells. Nucleic Acids Research 24: 4242–4248. 8932379 10.1093/nar/24.21.4242 PMC146228

[52] Montecucco A, Biamonti G (2007) Cellular response to etoposide treatment. Cancer Letters 252: 9–18. 17166655 10.1016/j.canlet.2006.11.005

[53] Adachi N, Iiizumi S, So S, Koyama H (2004) Genetic evidence for involvement of two distinct nonhomologous end-joining pathways in repair of topoisomerase II-mediated DNA damage. Biochemical and Biophysical Research Communications 318: 856–861. 15147950 10.1016/j.bbrc.2004.04.099

[54] Adachi N, Suzuki H, Iiizumi S, Koyama H (2003) Hypersensitivity of Nonhomologous DNA End-joining Mutants to VP-16 and ICRF-193. Journal of Biological Chemistry 278: 35897–35902. 12842886 10.1074/jbc.M306500200

[55] Schumacker PT (2006) Reactive oxygen species in cancer cells: Live by the sword, die by the sword. Cancer Cell 10: 175–176. 16959608 10.1016/j.ccr.2006.08.015

[56] Rashad AE, Mahmoud AE, Ali MM (2011) Synthesis and anticancer effects of some novel pyrazolo[3,4-d]pyrimidine derivatives by generating reactive oxygen species in human breast adenocarcinoma cells. European Journal of Medicinal Chemistry 46: 1019–1026. 21315495 10.1016/j.ejmech.2011.01.013

[57] Hug H, Strand S, Grambihler A, Galle J, Hack V, et al (1997) Reactive Oxygen Intermediates Are Involved in the Induction of CD95 Ligand mRNA Expression by Cytostatic Drugs in Hepatoma Cells. Journal of Biological Chemistry 272: 28191–28193. 9353266 10.1074/jbc.272.45.28191

[58] Armstrong JS, Steinauer KK, Hornung B, Irish JM, Lecane P, et al (2002) Role of glutathione depletion and reactive oxygen species generation in apoptotic signaling in a human B lymphoma cell line. Cell death and differentiation 9: 252–263. 11859408 10.1038/sj.cdd.4400959

[59] Franco R, Cidlowski JA (2009) Apoptosis and glutathione: beyond an antioxidant. Cell Death & Differentiation 16: 1303–1314. 19662025 10.1038/cdd.2009.107

[60] Gantchev TG, Hunting DJ (1997) Enhancement of etoposide (VP-16) cytotoxicity by enzymatic and photodynamically induced oxidative stress. Anticancer Drugs 8: 164–173. 9073312 10.1097/00001813-199702000-00007

[61] Vara JÁF, Casado E, de Castro J, Cejas P, Belda-Iniesta C, et al (2004) PI3K/Akt signalling pathway and cancer. Cancer Treatment Reviews 30: 193–204. 15023437 10.1016/j.ctrv.2003.07.007

[62] Sourbier C, Lindner V, Lang H, Agouni A, Schordan E, et al (2006) The Phosphoinositide 3-Kinase/Akt Pathway: A New Target in Human Renal Cell Carcinoma Therapy. Cancer Research 66: 5130–5142. 16707436 10.1158/0008-5472.CAN-05-1469

[63] Horiguchi A, Oya M, Uchida A, Marumo KEN, Murai M (2003) Elevated Akt Activation and Its Impact on Clinicopathological Features of Renal Cell Carcinoma. The Journal of Urology 169: 710–713. 12544348 10.1097/01.ju.0000038952.59355.b2

