# Supplementary material for: NPRL-Z-1, as a New Topoisomerase II Poison, Induces Cell Apoptosis and ROS Generation in Human Renal Carcinoma Cells
Source: PLoS One. 2014 Nov 5;9(11):e112220. doi: 10.1371/journal.pone.0112220 (PMC4221609; doi:10.1371/journal.pone.0112220)
Supplement: File S1 — Supporting Figures. Figure S1. Expression of TOP2α or TOP2β in A498, ACHN, and A549 cells. Three human cancer cell lines (A498, ACHN, and A549) were seeded overnight, harvested, and prepared for detection of TOP2α or TOP2β expression via western blotting. Figure S2. Effects of etoposide on cell cycle distribution in A498 cells. Cells were incubated with vehicle or various concentrations of etoposide for 24 h and detected cell cycle distribution by flow cytometry. Figure S3. Effects of NPRL-Z-1 in ACNH cells. (A) NPRL-Z-1 induced PARP cleavage. (B) NPRL-Z-1 induced DNA checkpoints activation. (C) Transfection of siTOP2α or siTOP2β could reverse NPRL-Z-1-induced cell death in ACHN cells. (DOCX) [file pone.0112220.s001.docx]

**Supporting Information**

Figure S1





Figure S2





Figure S3
